# Amelioration of NaCl stress on germination, growth, and nitrogen fixation of *Vicia* *faba* at isosmotic Na–Ca combinations and *Rhizobium*

**DOI:** 10.1007/s00425-024-04343-z

**Published:** 2024-02-10

**Authors:** Amal W. Danial, Refat Abdel Basset

**Affiliations:** Botany and Microbiology Department, Faculty of Science, Assiut University, Assiut, 71516 Egypt

**Keywords:** Faba bean, Growth conditions, Metabolites, Na–Ca combinations, Nitrogen fixation, Salinity, *Rhizobium leguminosarum*, *Vicia faba*

## Abstract

**Main conclusion:**

**The Na**^**+**^**/Ca**^**2+**^** ratio of 1/5 ameliorated the inhibitory action of NaCl and improved the germination and growth of ***Vicia faba*. **Addition of Rhizobium also enhanced nodulation and nitrogen fixation.**

**Abstract:**

Casting light upon the impact of salinity stress on growth and nitrogen fixation of *Vicia faba* supplemented with *Rhizobium* has been traced in this work. How Ca^2+^ antagonizes Na^+^ toxicity and osmotic stress of NaCl was also targeted in isosmotic combinations of NaCl and CaCl_2_ having various Na^+^:Ca^2+^ ratios. Growth of *Vicia* *faba* (cultivar Giza 3) was studied at two stages: germination and seedling. At both experiments, seeds or seedlings were exposed to successively increasing salinity levels (0, 50, 100, 150, and 200 mM NaCl) as well as isosmotic combinations of NaCl and CaCl_2_ (Na^+^:Ca^2+^ of 1:1, 1:5, 1:10, 1:15, 1:18, and 1: 20), equivalent to 150 mM NaCl. Inocula of the local nitrogen-fixing bacteria, *Rhizobium leguminosarum* (OP715892) were supplemented at both stages. NaCl salinity exerted a negative impact on growth and metabolism of *Vicia faba*; inhibition was proportional with increasing salinity level up to the highest level of 200 mM. Seed germination, shoot and root lengths, fresh and dry weights, chlorophyll content, and nodules (number, weight, leghemoglobin, respiration, and nitrogenase activity) were inhibited by salinity. Ca^2+^ substitution for Na^+^, particularly at a Na/Ca ratio of 1:5, was stimulatory to almost all parameters at both stages. Statistical correlations between salinity levels and Na/Ca combinations proved one of the four levels (strong- or weak positive, strong- or weak negative) with most of the investigated parameters, depending on the parameter.

## Introduction

Food, fodder, and energy are currently insufficient and magnified further by the increasing population all over the globe. Such utilizations of plants are linked to water availability as a detrimental factor in agriculture and crop production. Freshwater is in shortage in many areas of the world and consequent to such limitation; sunlight utilization by plants is inefficient and accounts for less than 1% (Hussein and Lambert 2020).

Concurrent to water deficit or drought, soil salinity is a worldwide naturally occurring barricade limiting the distribution of natural flora and hampering growth and productivity of agricultural crops. Approximately, 19.5% of all irrigated land and 2.1% of dry land is affected by salt stress (FAO 2021), continuing to increase because of mishandled irrigation (Shrivastava and Kumar 2015). In arid and semi-arid regions, the salinization process occurs because of inadequate amounts of precipitation along with high evaporation for considerable leaching (Dai et al. 2011). Besides, irrigation using wastewater resulted in high salt concentrations of N, P, and K in the topsoil, which decrease within depth, and the increase of salts in topsoil was proportional to its content in the wastewater used (El-Zohri et al. 2014; Wang et al. 2021). Global warming is also enhancing soil drought and, thus, salinization as a result of enhanced evapotranspiration, which imposes an additive burden on soil structure and plants, e.g., stomata are closed and their photosynthetic gas exchange with the atmosphere (CO_2_ uptake and O_2_ evolution) is inhibited.

Salinity exerts three types of stress: ion toxicity (of Na^+^ and Cl^−^), water deficit stress, and ion imbalance stress due to disturbed uptake of key elements (e.g., N and P); these collectively inhibit plants growth and productivity. Saline conditions lead to the production of reactive oxygen species (ROS) in chloroplasts, mitochondria, as well as in the apoplastic space (Juan et al. 2021) causing membrane peroxidation, ion leakage, and damage to nucleic acids, and cellular structure and ultimately reduces the quality and total yield of the affected crop. Lotfi et al. (2022) recorded increased K content in drought-stressed wheat plants, which plays a role in osmotic adjustment.

High salt can directly impair the biological nitrogen fixation (BNF) via impairing the interactions between *Rhizobium* and the host plant inhibiting nodule formation (Singleton and Bohlool 1984; Zahran 1999) and indirectly affecting the symbiosis by reducing the growth of the host plant. BNF and legumes are crucial not only for agriculture and crop yield but also for the environment, at least in terms of CO_2_ uptake and O_2_ release. Otherwise, the use of N fertilizers has degraded huge land extensions around the world and BNF is required to replace tons of synthetic fertilizers (Cassman and Dobermann 2022). Ca^2+^ deficiency is a typical feature of salt stress; the addition of Ca^2+^ to legumes grown under high NaCl concentrations had positive effects on nitrogen fixation (El-Hamdaoui et al. 2003).

Counteracting soil salinity is urgent in many countries having salinity-affected soils and limited freshwater resources; their population increases and actually suffering food shortage. Attempts conducted to overcome salinity-related adverse effects are numerous and diverse including repair of soil factors, adopting relevant halotolerant/halophytic plants, phytohormones, genetic manipulations (transgenic plants), etc. In this concert, calcium has long been used to overcome the adverse effects of numerous types of stresses, which widely vary in effects and nature (Abdel-Basset and Issa 1994; Abdel-Basset and Matsumoto 2008; Briffa et al. 2020). In this respect, Cramer et al. (1990) reported mitigation by calcium of progressively inhibited root elongation and osmolality of two corn (*Zea mays* L.) cultivars differing in their salt tolerance. Also, Ahmed et al. (1989) has found that certain Na–Ca combinations enhanced growth of the salinized cultures of the green alga *Chlorella* *vulgaris*. The maxima of cell number, dry matter and pigments, with the lowest value of respiration and the stress marker proline were recorded at an optimal Na^+^/Ca^2+^ ratio of 13.5. The calcium-induced alleviation of the harmful effects of NaCl may be due to the reduced uptake of Na and to the subsequent elevation of water content of hypocotyls and cotyledonary of regenerated tomato shoot under Na–Ca combination (El-Enany et al. 2001). The utilization of the tissue culture technique to derive cell lines tolerant to NaCl stress is one approach to the improvement of salt tolerance in tomatoes (Cuartero et al. 2006). It has been reported that increasing calcium concentration of the culture medium increases the frequency of somatic embryogenesis in salinized carrot and the organogenesis of shoots and roots (Dogan 2020). Calcium acts both at the intracellular level (Poovaiah and Reddy 1987) and at the apoplast level as an ion responsible for membrane stability, ion transport, and wall rigidity. It has also been reported that Ca is essential for K/Na selectivity.

Subsequently, the main objective of this work was to elucidate the potential ameliorative role played by calcium to diminish the negative impacts exerted by salinity in *Vicia faba*. Salinity is an inherent intrinsic characteristic in many areas of the world. Furthermore, most of the water on earth is saline. The case study plant *V. faba* was studied at two stages of growth: germination for 7 days in Petri dishes as well as seedlings in soil pots (45-day-old plants). Germination, fresh and dry mass, nodulation, chlorophyll, nitrogenase activity, leghemoglobin and respiration were assessed as measurables to the calcium-induced ameliorations in the salinity-strained *Vicia faba* plants*.* The insight of the herein study plan included early stages of plant development (seed germination and seedling growth), which is indicative for plant performance at its older stages (seed germination and seedling growth). Unique calcium ameliorative properties, ascribed to its ubiquitous interferences in cellular structures and metabolism, were also monitored. The case study plant, *Vicia faba*, is a major legume in Egypt; its importance as food and fodder are documented. It also may be useful in land reclamation, as it forms nitrogen-fixing nodules, which are important to overcome one of the major problems of salt-affected soils, i.e., nitrogen limitation and the subsequent need of excessive synthetic fertilizers. Remediation of soil salinity problems by calcium application at such laboratory work may be modeled to cultivable areas in Egypt and elsewhere in the world.

## Materials and methods

### Germination and seedling experiment

Seeds of *Vicia faba* (Giza 3) were obtained from the Faculty of Agriculture, Assiut University, Assiut, Egypt, and germinated in the presence of successively increasing NaCl-salinity levels in addition to various Na^+^/Ca^2+^ ratios. Germination was performed in 3 replicates in a Petri dish experiment in a growth chamber at 25 °C and followed for 7 days and terminated upon full germination of control seeds.

### Treatments

Four levels of salinity (50, 100, 150, and 200 mM NaCl) and Na–Ca combinations having Na^+^/Ca^2+^ ratios of 1:1, 1:5, 1:10, 1:15, 1:18, and 1:20, iso-osmotically equivalent to 150 mM NaCl, were applied to the pot grown plants same as in germination test in Petri dishes.

The Na^+^/Ca^2+^ ratios were calculated according to the following equation:

$$XNaCl=\frac{2\alpha \beta }{3+2\alpha },$$

where 2 and 3 are constants represent the number of dissociating ions from NaCl and CaCl_2_, respectively; α is the reciprocal of a certain (needed) Na^+^/Ca^2+^ ratio and β is the number of ions at a certain (needed) molarity of NaCl (= mM*2). “X” is the sum number of Na^+^, and Cl^−^ ions at a certain molar combination of NaCl and CaCl_2_.

Concentration of NaCl (Y mM) = X/2,

Concentration of CaCl_2_ (Z mM) = (β– X)/3.

a) Germination assessment

Seed germination was assessed by the following parameters:

–Germination percentage (GP):

GP = (number of germinated seeds ÷ total number of seeds sown) × 100,

–Germination index (GI): GI = Σ(Gt/Tt),

where Gt is the number of seeds germinated on day t, and Tt is the number of days (Hakim et al. 2010; Keshavarizi and Mohammed 2012).

Mean germination time (MGT):

–MGT = Σ(Ti × Ni)/ΣNi,

where Ni is the number of newly germinated seeds at time Ti (Ruan et al. 2002).

b) Seedling assessment

Seedling growth was assessed by fresh weight, dry weight and seedling vigor index (SVI). For seedling growth, ten seedlings were randomly selected from each Petri dish at the end of the germination period. After selection, shoot length (SL) and root length (RL) were measured in cm. Seedlings’ fresh weight (FW) and dry weight (DW), after drying the samples in an oven for 72 h at 80 °C, were recorded. In addition, SVI (Mahender et al. 2015) was assessed as follows:

SVI = mean germination percentage × mean seedling length.

### Experimental set-up for plant sowing

Seeds of faba bean (*Vicia faba* L. Giza 3 cultivar) were surface sterilized by ethanol (70% for 30 s), NaOCl (5% for 3 min), and washed 5 times with sterilized water. Ten sterilized seeds were then planted in soil pots and sown for 60 days at plastic pots of 7 kg capacity; each pot contained 5 kg of sand/clay (1:2) soil from around Assiut city area. Seedlings were thinned to four plants per pot of comparable height and vigor at 7 days after planting. Table 1 represents the soil mixture analysis. The experiment was laid out randomly with three replicates in the greenhouse and the pots were rotated regularly on the benches to equalize the effect of sunlight intensity at the different times of the day. Plants were irrigated with distilled water up to the field capacity every other day over the 60 days of growth. Inoculation with bacteria was done twice after 15 and 30 days from sowing the seeds. The growth experiment was conducted in the botanical garden, Department of Botany and Microbiology, University of Assiut during the growing season of *Vicia faba* (November–February) under natural conditions of light, temperature, and humidity. Plants were harvested for analysis at 15, 30, 45, 60 days after planting. The experimental design and analytical procedure were always conducted in three replicates.

**Table 1:** Soil analysis (sand/clay 1/2) at which *Vicia faba* was grown

Soil parameter	Value
pH	7.9
Electrical conductivity, mM.cm^−1^	0.21
Organic carbon, %	0.65
Available nitrogen, kg ha^−1^	185
Available phosphorus (Olsen’ P), kg ha^−1^	8.76
Available potassium, kg ha^−1^	610
Available sulfur, kg ha^−1^	11.73

Seeds in Petri dishes and seedlings in soil pots were inoculated with a salinity-tolerant strain of *Rhizobium leguminosarum*; the herein-newly isolated strain from bean nodules cultivated in salinized soil. Control pots received neither of the above treatments nor the bacterium.

### Isolation of *Rhizobium* strain from *Vicia faba* nodules

Nodules were detached from *V. faba* roots, washed with sterile water followed by surface sterilization with 95% alcohol and again with sterile water. The nodules were, then, surface sterilized with 0.1% sodium hypochlorite for 2–3 min and again washed for at least 10 times with sterile water. The nodules were transferred into culture tubes half filled with sterile water and crushed with a sterile glass rod to obtain a milky bacterial suspension. After serial dilutions, the suspension was streaked on yeast extract mannitol agar (YEMA) plates and incubated for 2–3 days at 28 °C. A single colony was taken from the agar plates and re-streaked on freshly prepared YEMA plates to obtain the pure culture (Gachande and Khansole 2011), which was grown in YEM broth to be used for DNA extraction.

### Molecular identification of rhizobia

The morphological, biochemical, and molecular characteristics of the isolate were used for strain characterization, according to Bergey’s Manual of Systematic Bacteriology (Brenner et al. 2005). The bacterial strain was identified according to the partial 1500 bp sequences of 16Sr RNA of the strains, and comparison in the GenBank databases. The total genomic DNA was extracted and purified from the bacterial samples. The primer set of F (5-AGA GTT TGA TCC TGG CTC AG-3) with a GC clamp and R (5-GGT TAC CTT GTT ACG ACT T-3) at the annealing temperature of 65 °C was used for the PCR amplification of the variable region of 16S rDNA from the purified genomic DNA. Then, PCR clean up to the PCR product was made using GeneJET™ PCR Purification Kit (Thermo Scientific). Loading to 4 µl from the PCR mixture was carried out to examine the PCR product on 1% agarose gel against 1 kb plus ladder (Fermentas). Finally, sequencing of the PCR product on GATC Company using ABI 3730xl DNA sequencer, forward and reverse primers was conducted. Sequence analysis was achieved by searching through the online databases using BLAST. The phylogenetic analysis was performed using MEGA 3.1 software. The phylogenetic tree was constructed by the neighbor joining method. The sequences obtained were compared with the available database sequences using a BLAST search. The sequence of the isolated strain was deposited in the Gen Bank.

### Organism and inoculation

The isolated strain was identified as *Rhizobium leguminosarum*, which was then grown in 250 ml Erlenmeyer flasks containing 100 ml yeast extract mannitol (YEM) broth (Somasegaran and Hoben 1985) in a shaker incubator for 3 days until reaching maximum turbidity (approximately 1 × 10^9^ cells ml^−1^). Bacterial inoculum population was estimated with plate dilution method and total count (Vincent 1970). Ten ml of the log phase bacterial culture was inoculated into each pot, watered regularly to maintain the soil at field capacity.

## Analytical methods

### Soil analysis

The used soil was prepared (sand:clay 1:2), air-dried, and soil samples (0–20 cm top layer) were analyzed before addition of salt treatments using standard procedures (Central lab, Faculty of Agriculture, Assiut University). In brief, soil pH was determined (HI 2216; Hanna, Smithfield, RI, USA). Available P was extracted using the Mehlich-3 and determined using the ammonium vanadate method and amount determined using a spectrophotometer (Mehlich 1984). Organic carbon was determined by Walkley and Black (1935) sulfuric acid–dichromate digestion followed by back titration with ferrous ammonium sulfate, whereas nitrogen was determined using the Kjeldahl method (Bremner and Mulvaney 1982).

### Plants

The soil was gently washed off the roots under a stream of running tap water; the nodules were then carefully removed from the roots, counted, and weighed. Roots and shoots were separated, and the fresh weight (FW) of each part was recorded for each plant. They were dried inside a paper envelope in an oven at 80 °C for 24 h, and the dry weights (DWs) were recorded.

Germinating seeds were counted, shoot and root lengths, fresh and dry mass were measured and the averages of 10 shoots or roots ± SE at each treatment are presented. Average nodule numbers of 10 plants ± SE were recorded.

### Metabolic pools

Chlorophylls (a and b) contents were assessed in ethanol extracts according to Arvola (1981), calculated and expressed as µg/ml according to Metzner et al. (1965).

In water extracts, reducing sugars were estimated as glucose equivalents according to Miller (1959) and soluble protein contents were estimated using the method of Lowry et al. (1951) using UV-120 spectrophotometer (MioTech, Hong Kong, China).

### Determination of leghemoglobin in nodule cytosol

One gram of fresh nodules was rinsed thoroughly with distilled water and immediately hand ground in an ice chilled mortar with 5 ml of distilled water. Nodule homogenates were filtered, and the filtrate was centrifuged at 500 g for 2 min to remove nodule debris. The resulting supernatant was centrifuged at 12,000 g for 15 min to sediment the bacteroids. Leghemoglobin levels in the supernatant, the ‘nodule cytosol’, were determined calorimetrically essentially as described by Larue and Child (1979) using Unico UV-2100 spectrophotometer. The colorimetric assay was standardized using freshly prepared Hemetrol reagent (solution of cyanmethemoglobin titrated exactly according to recommendations of BioMerieux (Marcy L'Etoile, France).

### Nitrogenase activity

Nitrogenase activity, as acetylene reduction, was determined in excised nodulated roots, using gas chromatograph (Thermo Scientific, TRACE GC Ultra). The roots were placed in 500 ml bottles sealed with a rubber septum; 50 ml of air were taken, and the same volume of acetylene gas was introduced into the bottle, incubated at 37 °C. Then, samples from the root nodules atmosphere in bottles were withdrawn and injected to the gas chromatograph. Afterward, nodules of each individual root were counted, and their fresh and dry mass were determined. A calibration curve was constructed using pure ethylene.

### Respiration (oxygen uptake measurements)

Respiration of *Rhizobium leguminosarum* was measured both in vitro (germination stage) as well as in vivo (nodules). The respiratory oxygen uptake (R) of *Rhizobium leguminosarum* was monitored daily (hroughout the germination period of 7 days) using a Clark type electrode computerized to an Oxygen Monitoring System (OMS, Hansatech Instruments Inc., donation from the Alexander von Humboldt Foundation Germany to R. Abdel Basset). Two milliliters of bacterial suspension was monitored at 25 °C for 15 min. In seedlings, respiration of excised nodules was also monitored; the rate was calculated and expressed as mmole O_2_ mg protein^−1^ h^−1^.

### Membrane stability index and percentage of membrane injury

Twenty leaf discs were placed into l00 ml flasks and washed thoroughly with three changes of deionized water to remove surface adhered electrolytes. Discs were then kept in thirty ml of deionized water for 24 h at 10 °C in the dark. Afterward, the flasks were warmed to 25 °C, shaken well and the electrical conductivity was measured. Following the conductivity measurements, the leaf tissues were autoclaved for 15 min to release all ions from the tissue, cooled to 25 °C and then the electrical conductivity was measured again. Cell membrane stability (CMS) and relative cell injury were calculated with the formula used by Nijabat et al (2020).

Cell membrane stability (%) = 1 − (E1/ E2) × 100,

where E1 and E2 are the electric conductivity before and after autoclaving, respectively.

Relative cell membrane injury (%) = [1 − (CMS2) / 1 − (CMS1) × 100],

where CMS1 is cell membrane stability for control samples, CMS2 is for salinity-treated samples.

### Statistical analysis

Each experiment was repeated three times and the mean values of three replicates ± SE (standard errors) are presented. Statistical analysis of the data was conducted using ANOVA one-way test (analysis of variance) by SPSS program version 21, and Duncan values were determined at 0.05 levels.

## Results

**Fig. 1:**
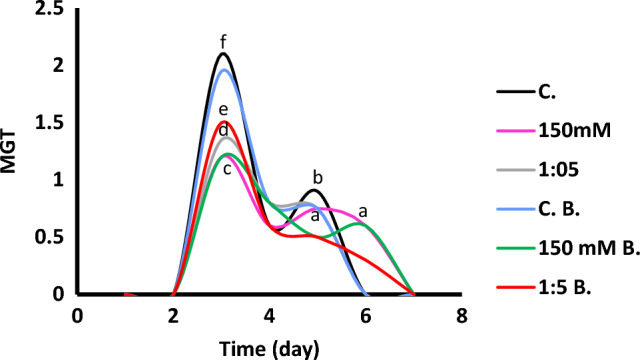
Mean germination time (MGT) of 7-day-old *Vicia faba* seeds after successively increasing NaCl levels and Na:Ca ratios. The ANOVA test was carried out using SPSS 21 comparisons among means (*n* = 3), at *P* = 0.05 level based on Duncan's multiple range test

In this work, *Vicia faba* seeds and seedlings were exposed to successfully increasing NaCl concentrations of 0, 50, 100, 150, and 200 mM as well as to various Na/Ca ratios of 1:1, 1:5, 1:10, 1:15, 1:18, and 1:20, iso-osmotically equivalent to 150 mM NaCl. *Vicia faba* displayed symptoms of negative impacts of salinity. At both plant growth stages (germination and seedlings), faba bean plants were negatively affected by increasing salinity level. Salinity reduced all germination aspects of *V. faba*; namely GP (germination percentage), germination index (GI), mean germination time (MGT) and seedling vigor index (SVI) (Table 2 and Fig. 1). The highest MGT was observed at control seeds and the lowest was found at 150 mM NaCl (Fig. 1). The addition of bacteria did not enhance MGT either at control or salinized (150 mM NaCl) seeds. However, Na^+^/Ca^2+^ ratio of 1:5 stimulated MGT and further by the addition of bacteria.

**Table 2:** Germination parameters of *Vicia faba* at control, 150 mM NaCl, and Na/Ca of 1:5 in presence or absence of *Rhizobium*

Treatments		GP	GI	SVI
Without bacteria	Control	100	9.553	1200
	150 mM	80	6.581	336
	1:5	90	7.201	1242
With bacteria	Control	100	9.300	1190
	150 mM	80	6.709	336
	1:5	95	7.267	1292

*GP* germination percentage (%), *GI* germination index, *SVI*  seedling vigor index

**Fig. 2:**
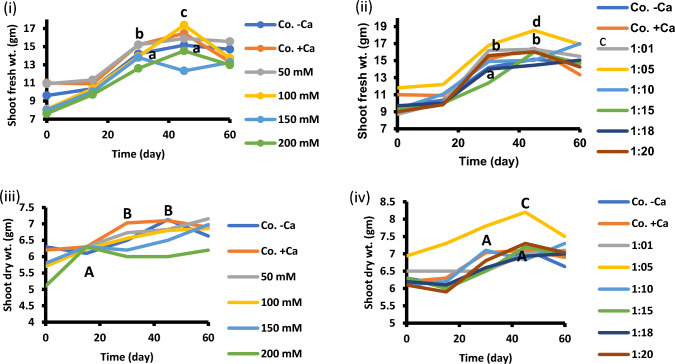
Shoot fresh and dry weight of 60-day-old *Vicia faba* seedlings at successively increasing NaCl levels (**i** and **iii**) and at Na: Ca ratios (**ii** and **iv**). The ANOVA test was carried out by using SPSS 21 comparisons among means. (*n* = 3); small letters (**a**–**d**) show significance in fresh weights at the different treatments while capital letters (**A**–**C**) refer to statistical differences among dry weights, at *P* = 0.05 level based on Duncan's multiple range test. Letters indicate significance between treatments at their maxima, where they are placed might represent more than one treatment.

**Fig. 3:**
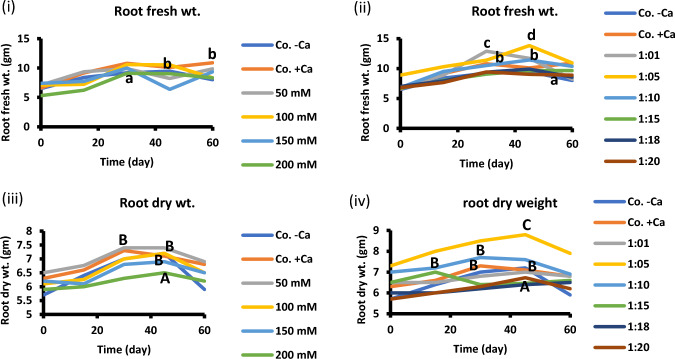
Root fresh weight of 60-day-old *Vicia faba* seedlings at successively increasing NaCl levels (**I** and **iii**) and at Na: Ca ratios (**ii** and **iv**). The statistics in Fig. 2 applies also to this figure

**Fig. 4:**
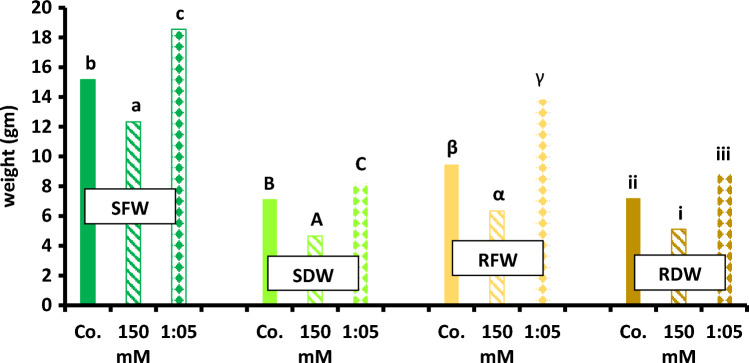
Maxima after 45 days of growth of variously treated *Vicia faba* plants at successively increasing NaCl levels and at Na: Ca ratios (only the concentration of 150 mM NaCl and the ratio of 1:5 are shown to conserve space). SFW is shoot fresh weight, SDW is shoot dry weight, RFW is root fresh weight and RDW is root dry weight. The letters (**a**, **b**, **c**) and (α, β, γ), and digits (1, 2, 3) and (i, ii, iii) refer to statistical differences among SFW, SDW, RFW and RDW, respectively; maxima are abstracted from Figs. 2 and 3 subjected to the ANOVA test carried out using SPSS 21 comparisons among means (*n* = 3)

**Fig. 5:**
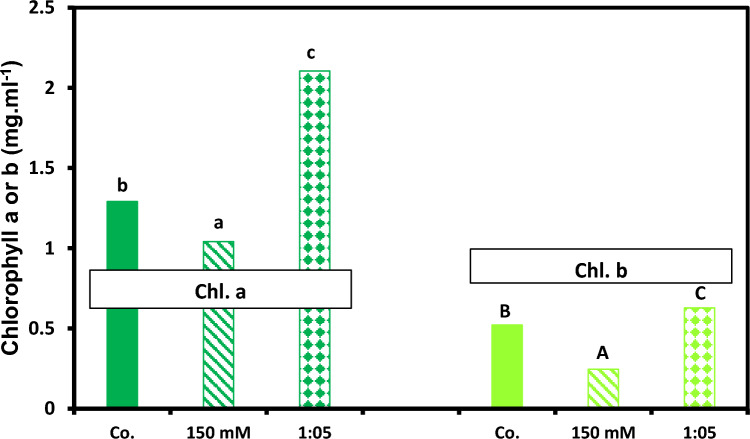
Maxima after 45 days of growth of variously treated *Vicia faba* plants at successively increasing NaCl levels and at Na: Ca ratios (only the concentration of 150 mM NaCl and the ratio of 1:5 are shown to conserve space). Chl.a, chlorophyll a; Chl.b, chlorophyll b. The statistics in Fig. 2 applies also to this figure

Seedling and plant growth are enhanced by addition of 1:5 Na/Ca treatment compared to control. Fresh and dry mass of shoots are improved significantly at 1:5 supplantation media (Fig. 2). Also, fresh and dry mass of root exhibited similar trend as those of the shoot in presence of 1:5 Na/Ca combination (Fig. 3). Figure 4 summarizes the data in Figs. 2 and 3. It presents the maximum results of growth criteria (fresh and dry mass) that were induced by a certain Na/ratio of 1/5. Figures 2 and 3 present the detailed treatments (salinity levels and Na–Ca combinations) as well as their results, from which the maximum values were selected and presented in Fig. 4. Also, Figs. 2 and 3 set a comparison between the optimum treatment (Na:Ca ratio of 1: 5) that led to the highest growth magnitude with the different salinity levels and other Na/Ca ratios. Chlorophyll content was stimulated at the same ratio of Na/Ca 1:5 (Fig. 5).

**Fig. 6:**
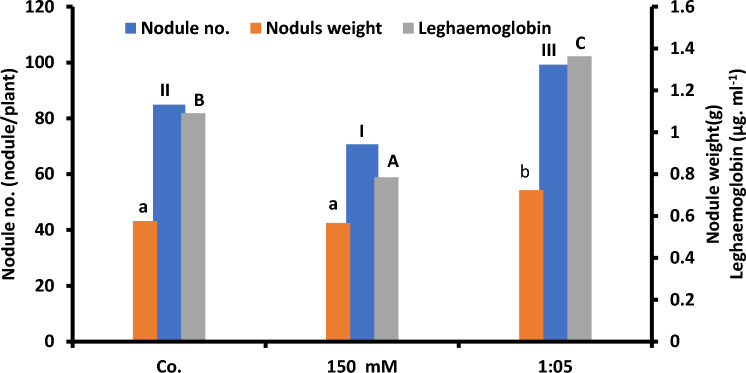
Maxima of nodule number, nodule weight, and leghemoglobin of 15-day-old *Vicia faba* plants variously treated at successively increasing NaCl levels and at Na:Ca ratios (only the concentration of 150 mM NaCl and the ratio of 1:5 are shown to conserve space. The statistics in Fig. 2 applies also to this figure

**Fig. 7:**
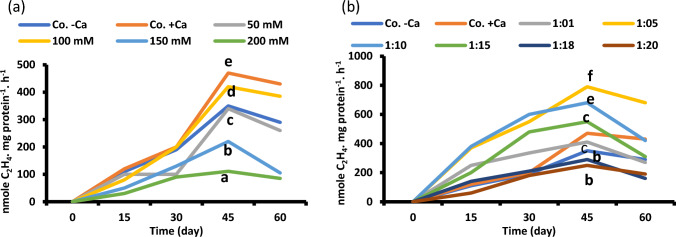
Nitrogenase activity at 60-day-old *Vicia faba* seedlings of successively increasing NaCl levels (**a**) and at Na: Ca ratios (**b**). The statistics in Fig. 2 applies also to this figure

Nodules number, nodules weight, and leghemoglobin were gradually inhibited by increasing salinity levels (Fig. 6a) and enhanced significantly up to their maximum levels at Na:Ca of 1:5 ratio (Fig. 6b). Nitrogenase activity followed, more or less, the same attitude using the same treatments (Fig. 7).

**Fig. 8:**
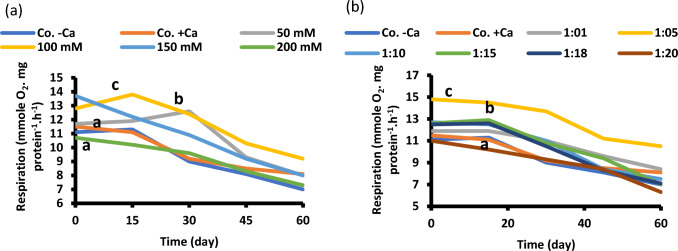
Respiration of 60-day-old *Vicia faba* seedlings (a) at successively increasing NaCl levels (**a**) and at Na: Ca ratios (**b**). The statistics in Fig. 2 applies also to this figure

Respiration rates (dark oxygen uptake) of faba seedlings were also inhibited by salinity and maximized by the same ratio (Fig. 8). Enhanced respiration by the optimum Na/Ca ratio of 1/5 indicates the divergence of the generated respiratory energy for growth, i.e., synchronization of energy production with growth.

**Fig. 9:**
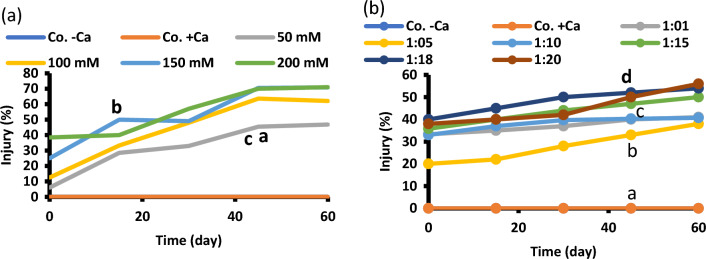
Percentage of membrane injury of 60-day-old *Vicia faba* seedlings at successively increasing NaCl levels (**a**) and at Na: Ca ratios (**b**). The statistics in Fig. 2 applies also to this figure

Membrane injury (Fig. 9) was elevated by increasing the level of NaCl. The least injury level was recorded at Na–Ca combinations, particularly at 1: 5. Membrane stability index, which is almost the opposite of membrane injury, was calculated as well; it was inhibited by increasing salinity level and improved by calcium (data not shown to conserve space). Minimum injury (ions and metabolites leakage) is in accordance with maximum growth (highest biomass fresh and dry weight).

Calcium ions were applied to both germination in petri dishes and growth in the soil pots as various Na/Ca ratios of 1:1, 1:5, 1:10, 1:15, 1:18, and 1:20, iso-osmotic to 150 mM NaCl. The partial substitution of Ca^2+^ for Na^+^ relieved NaCl stress; particularly, the ratio of Na/Ca 1:5 resulted in the highest relief of seed germination, root length, shoot length, fresh weight of root and shoot, and dry mass of root and shoot, respiration (Table 3).

**Table 3:** The highest seed germination, length, fresh, dry weight, and respiration of 7-day-old *Vicia faba* shoots and roots at control, 150 mM NaCl and Na/Ca of 1:5 in the presence or absence of bacteria

	without bacteria			with bacteria		
	Control	150 mM	1:05	Control	150 mM	1:05
Number of germinated seeds	10e	8d	9e	10e	8d	9.5e
Shoot length (cm)	5.65f	1.80d	7.05 g	5.40f	1.85d	6.75f
Root length (cm)	6.40 g	2.40e	6.80 g	6.55 g	2.40e	6.95 g
Shoot fresh weight (g)	5.10e	2.05c	2.80d	5.65e	2.25 cd	2.80d
Root fresh weight (g)	6.75f	4.15e	6.20f	7.20f	4.30e	6.90f
Shoot dry weight (g)	1.14e	0.41bc	0.66d	1.18e	0.66d	0.95e
Root dry weight (g)	1.25f	0.66d	0.90e	1.50f	0.74d	0.87e
Respiration* (mM O_2_ 10^3^ cell^−1^ h^−1^)	16c	10b	20d	23d	15bc	30f

The results in this table are abstracted from the complete survey at 0, 50, 100, 150, and 200 mM NaCl) as well as isosmotic combinations of Na–Ca combinations (Na: Ca 1:1, 1:5, 1:10, 1:15, 1:18, and 1: 20 of NaCl and CaCl_2_), equivalent to 150 mM NaCl. The statistics in Fig. 2 applies also to this table

^*^Exceptionally, the highest respiration presented is for 5-day-old, germinated seeds

**Fig. 10:**
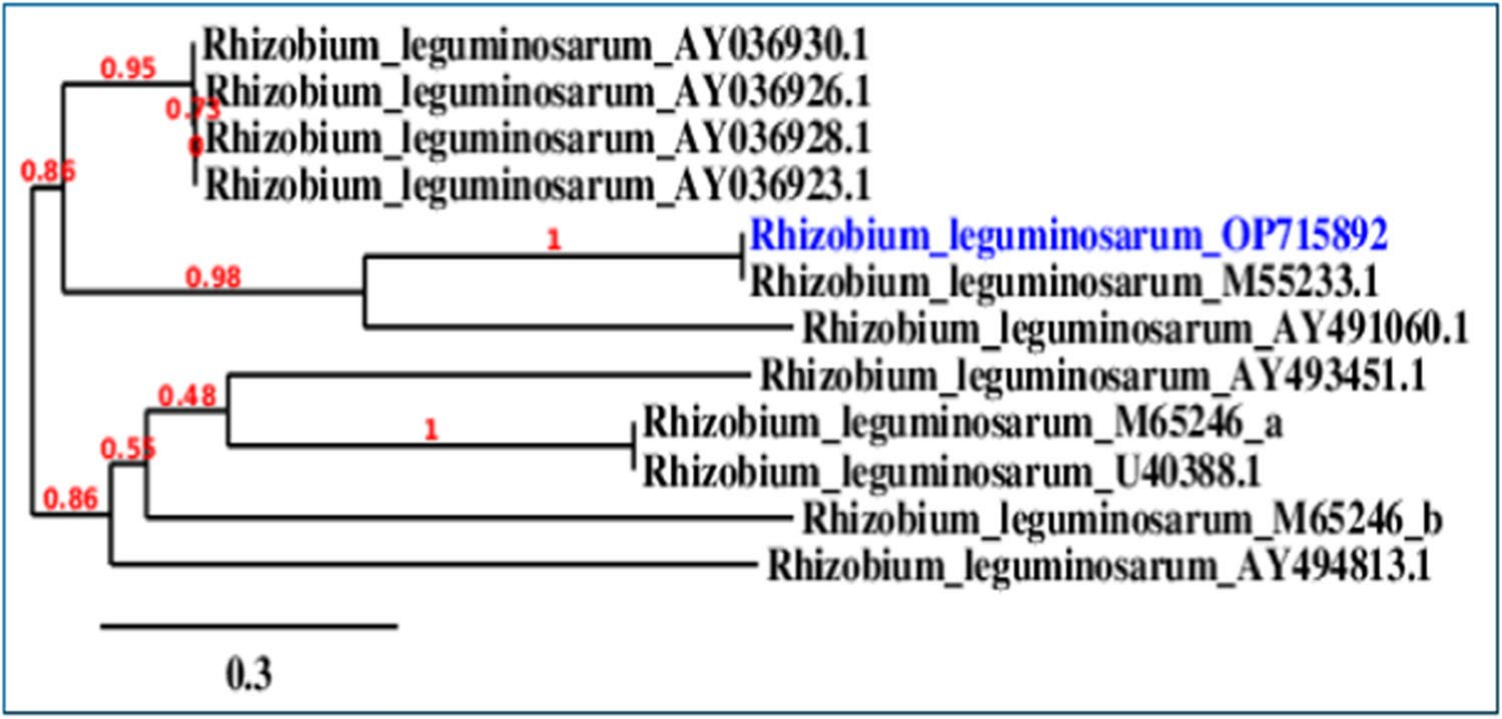
Phylogenetic tree on the basis of patterns and genetic relationship of the case-studied *Rhizobium leguminosarum* OP715892. (For interpretation of the references to color in this figure legend, the reader is referred to the Web version of this article.)

The bacterial isolate from faba nodules was identified as *Rhizobium leguminosarum* with similarity 100% and the sequence was deposited in the Gene Bank under the accession number of OP715892. Figure 10 shows the phylogenetic tree of the studied isolate, *Rhizobium leguminosarum* (OP715892).

- The correlations between all the assessed criteria in *Vicia faba* with age, salinity level or sodium/calcium combinations, at which plants grew, were statistically analyzed (Table 4). These criteria included fresh and dry weight of shoots and roots, chlorophyll content, Chl a/Chlb ratios, nodule number, nodule content of leghemoglobin, nodule respiration, and their nitrogenase activity. The statistical analysis between treatments (salinity levels and Na/Ca combinations) with the studied parameters revealed one of four statistical correlations: strong positive, weak positive, strong negative or weak negative, depending on each of the parameters. In summary, the salinity level of 150 mM NaCl induced a strong positive correlation between shoot fresh and dry weight while root fresh and dry weight, nodule number and chlorophyll induced weak positive correlation with time. Nodule weight and respiration induced a strong negative correlation while leghemoglobin and nodule weight induced weak negative correlation with time. The Na/Ca ratio of 1/5 induced a strong positive correlation between shoot fresh and dry weight whereas root fresh and dry weight, nodule number and chlorophyll induced weak positive correlation with time.
- Although, the other treatments induced correlations with the measured criteria.
- The statistical analysis revealed statistical correlations between most of the studied parameters with one or both of the treatments (salinity levels or Na/Ca combinations) at one of four correlation levels (strong/ weak positive, strong/ weak negative), specific to each parameter.

**Table 4:** Correlation between various growth criteria with salinity and time of *Vicia faba* plants grown for 60 days at successively increasing levels of NaCl salinity and Na/Ca ratios isoosmotic to 150 mM NaCl

		Shoot fresh wt	Shoot dry wt	Root fresh wt	Root dry wt	Nodule number	Nodule wt	Leghemoglobin	Nodule Respiration	Chl Chlorophyll a	Chlorophyll b
Salinity	0	− 0.848*	− 0.885*	− 0.321	− 0.114	− 0.974**	− 0.335	− 0.925**	0.221	− 0.909*	− 0.834*
	15	− 0.734	0.469	− 0.845*	− 0.819*	− 0.911*	− 0.633	− 0.679	− 0.060	− 0.813*	− 0.907*
	30	− 0.838*	− 0.860*	− 0.325	− 0.830*	− 0.903*	− 0.602	0.571	0.178	− 0.610	− 0.805
	45	− 0.443	− 0.965**	− 0.312	− 0.778	− 0.970**	− 0.639	− 0.545	0.179	− 0.838*	− 0.908*
	60	− 0.557	− 0.461	− 0.370	− 0.153	− 0.792	− 0.166	− 0.002	0.043	ND	
Time	Co. -Ca	0.907*	0.685	0.548	0.288	0.117	− 0.867*	− 0.887*	− 0.961**	0.416	− 0.352
	Co. + Ca	0.651	0.821	0.852	0.599	0.252	− 0.676	− 0.625	− 0.967**	0.583	− 0.141
	50 mM	0.897*	0.969*	0.575	0.570	0.589	− 0.380	− 0.694	− 0.808	0.703	− 0.582
	100 mM	0.810	0.957*	0.532	0.582	0.529	− 0.846	− 0.482	− 0.894*	0.505	− 0.662
	150 mM	0.849	0.937*	0.658	0.626	0.164	− 0.879*	− 0.126	− 0.999**	0.428	− 0.700
	200 mM	0.885*	0.631	0.809	0.728	0.380	− 0.953	0.181	− 0.983**	0.501	0.072
Time	Co. -Ca	0.907*	0.685	0.548	0.288	0.117	− 0.867	− 0.887*	− 0.961**	0.416	− 0.352
	Co. + Ca	0.651	0.821	0.852	0.599	0.252	− 0.676	− 0.625	− 0.967**	0.583	− 0.141
	1:1	0.855	0.886*	0.350	0.802	0.451	− 0.555	− 0.858	− 0.961**	0.521	− 0.724
	1:5	0.858	0.665	0.664	0.546	0.406	− 0.443	− 0.643	− 0.957*	0.597	− 0.815
	1:10	0.967**	0.895*	0.809	0.089	0.362	− 0.860	− 0.638	− 0.967**	0.547	− 0.369
	1:15	0.914*	0.818	0.944*	− 0.225	0.554	− 0.785	− 0.781	− 0.955*	0.580	− 0.517
	1:18	0.930*	0.937*	0.697	0.971**	0.766	− 0.517	− 0.831	− 0.967**	0.560	− 0.599
	1:20	0.801	0.856	0.803	0.718	0.622	− 0.599	− 0.317	− 0.976**	0.677	− 0.259

The positive sign ( +) indicates positive correlations and the negative sign (−) indicates negative correlations while one star (*) indicates weak correlation value (< 0.7) and two stars (**) indicate strong correlation (≥ 0.7); no star indicates no correlation

## Discussion

In this work, growth of *Vicia faba* was stressed by NaCl salinity at two stages of growth: germination and seedling. In this context, the reaction of plants to salinity in germination and seedling stages seems potential determinants for plants salt tolerance and the production of appropriate crop yields in saline conditions. In accordance with the above statement, Krishnamurthy et al. (2007) concluded that germination and seedling traits can be reliable indices for the final plant performance at salinity conditions. Seed germination was regarded as an important and susceptible stage of plant growth because the duration of this phase determines seedling establishment and future plant growth (Hakim et al. 2010). Tolerance to salt stress at the germination stage and at seedling emergence determines better plant establishment in saline soils (Bojovic et al. 2010; Keshavarizi and Mohammed 2012)**.** Salinity inhibited growth of *Vicia faba*, while the partial substitution of Ca^2+^ for Na^+^ ameliorated the adverse effects of salinity at both stages of growth. Gemination rate index, seedling vigor index (SVI), germination percentage (GP) and mean germination time (MGT) were all inhibited by salinity but enhanced by calcium addition. Shoot-, root length, fresh and dry weight similarly responded. Our results in *Vicia faba* agree with those of *Sorghum* (Geressu and Gezahagn 2008; Dehnavi et al. 2020). Germination and emergence stages in *sorghum* development were the most informative stages of the plant’s lifecycle to evaluate the effect of salinity (Krishnamurthy et al. 2007). Responses in *Sorghum* varied depending on the genotype (Nimir et al. 2014, 2015; Ali et al. 2020). Also, increasing salinity significantly reduces germination percentage and rate, root and shoots lengths, and fresh and dry weights of the exposed plants (Shrivastava and Kumar 2015). In addition, Mbinda and Kimtai (2019) reported that salinity substantially affects all traits associated with germination and early seedling growth of *Sorghum*, depending on the variety used and level of salinity stress applied. Salinity has also negative correlation with germination percentage (GP), germination index (GI), and VIG of *Imperata cylindrica* (Rehman et al. 2000).

Chlorophyll contents in faba leaves were also lowered as salinity was increased while was enhanced by calcium addition. Chlorophyll plays a crucial role in the absorption and transmission of light quanta and chlorophyll concentration is an indicator of a plant's capacity to utilize light for photosynthesis (Zhang et al. 2018; Danial et al. 2023). Under saline conditions the reduction of seedling shoot, and root lengths FW, and DW is a common phenomenon in many plants. The effects of salinity on root length of legume plants were more drastic than on shoot length, which might be due to the effect of NaCl being more inhibitory on root growth than on shoot growth (El-Beltagi et al. 2022; Alnefaie et al. 2023). Na^+^ accumulates more in roots than in shoots, e.g., in *Sorghum* plants (Tester and Davenport 2003). Similarly, faba plants responded. Roots are the first organs exposed to salinity and are in direct contact with the soil, absorbing water and salts from the soil and supplying it to the shoot (Asaadi 2009). However, the reduction in FW and DW may be due to the toxic effect of Na^+^ on photosynthesis (Kawasaki et al. 1983). In fact, salinity influences the germination process by several modes of action; namely, less water uptake, Na^+^ and Cl^−^ ion toxicity, disturbed nutrient uptake, enzymatic and subsequent metabolic disturbances. First, salinity reduces the imbibition of water by seeds due to the lower water potential of germination media, i.e., osmotic or pseudo-drought stress. Outer osmotic stress inhibits water uptake and may further drain water from inside to the outer hypertonic medium. Delayed water absorption, thus, reduces germination (Farhoudi and Tafti 2011; Dehnavi et al. 2020; Misra and Gupta 2020). In this respect, NaCl inhibited seed germination due to the high osmotic potential, specific ion toxicity (Na^+^ and Cl^−^) and inhibited maintenance of nutrient levels essential for plant growth such as NO_3_^−^ (Chien et al. 2009). In the present work, water content of faba shoots and roots was decreasing with the increase of NaCl level, which is in accordance with the above interpretations of salinity-inhibited water uptake (Zheng et al. 2022; Cen et al. 2023). On the other side, as NaCl ions passively accumulate inside cells, salinity causes disruption of enzymatic activities, which subsequently leads to major changes in plants during germination, such as altering the metabolism of nucleic acid and protein (Dantas et al. 2007), disturbing the hormonal balance (Ryu and Cho 2015), and reducing the use of seed reserves (Othman et al. 2006). Additionally, it seems that by inducing disturbance of the metabolic process, salinity increases phenolic compounds, which can reduce germination (Ayaz et al. 2000). Specifically, salinity reduces intercellular CO_2_ concentration and then photosynthesis rate by stomatal closure (Kaymakanova and Stoeva 2008). In addition, salinity exerts a negative effect on the ultrastructures of cells, tissues, and organs (Koyro 2002). Ultimately, salinity is limiting to root emergence and seedling growth (Krishnamurthy et al. 2007; Abari et al. 2011; Bilgili et al. 2011). Collectively, salinity inhibits seeds germination by multiple and diverse modes of action, which disturbs homeostasis of various nutrients and metabolic processes. Furthermore, various seeds internal factors, such as coat properties, age, polymorphism, dormancy, seedling vigor; and external factors, such as temperature, light, water, and gases, can affect seed germination under saline conditions (Wahid et al. 2011). Numerous studies reported that, under saline conditions, genotypes which maintain higher germination rates are salt tolerant and produce higher biomass and yield (Dehnavi et al. 2020). It has been argued that retention of Na^+^ ions occurred in roots of salt tolerant genotypes (Assaha et al. 2017). Otherwise, Chien et al. (2009) reported that tolerant genotypes have lower uptake of Na^+^ than sensitive genotypes do.

All biological nitrogen fixation parameters (BNF) were inhibited as the salinity level was increased and ameliorated upon addition of calcium; these parameters include nodule number, nodule weight, leghemoglobin, nitrogenase activity. A novel isolate of *Rhizobium leguminosarum* (OP715892) was supplemented to faba plants for nodulation; it has been inhibited by salinity and enhanced by calcium in terms of the assessed BNF parameters. Although this bacterial strain was inhibited by salinity, it can be inferred that it is a halotolerant one as it survived salinity and its BNF parameters continued and were supportive for the bean plants’ growth, although in defected rates. Plants are usually less tolerant to stress than the microsymbiont (Zahran 1991)**.** The addition of a Ca^2+^ supplement can recover nodulation inhibited by salt (Etesami and Adl 2020). Moreover, salinity inhibits nitrogen fixation in nodules by the deficiency of important nutrients, which can be recovered by a balanced Ca^2+^ nutrition (El-Hamdaoui et al. 2003). The role of Ca^2+^ seems related to the protection of nitrogenase enzyme complex from oxygen (Sabra et al. 2000). Similar studies should establish the best Ca^2+^ concentration for each type of legume that ensures the success of symbiosis and plant development in salinity (El-Hamdaoui et al. 2003). Since early times, calcium is known as a requirement at the early stages of infection events (Munns 1970) to increase the number of nodules (Lowther and Loneragan 1968), and for symbiotic nitrogen fixation (Banath et al. 1966). Purified nodulation (Nod) factors elicit several physiological responses in susceptible root hair cells, such as calcium influx, membrane depolarization, and rhythmic Ca^2+^ oscillations (spiking) in and around the nucleus (Oldroyd and Downie 2004). Martins and Livina (2019) supported the idea of two symbiotic Ca^2+^ signals: a Ca^2+^ flux at the root hair tip and nuclear Ca^2+^ oscillations with different frequencies characteristic of different symbiotic phenomena. Ca^2+^ spiking is a key component in the activation of zone I nodulation via root hair curling (RHC) invasion (Capoen et al. 2009). Nitrogen fixers trigger faster Ca^2+^ oscillations than during RHC nodulation. Modulation of the ethylene or JA levels slowed down the Ca^2+^ spiking frequency and stimulated RHC invasion but was incompatible with nodule development. Bioimaging suggests that there are distinct Ca^2+^ signals predominantly associated with each region: a Ca^2+^ influx at the root hair tip region, which is thought to be involved in the formation of an infection thread, and nuclear Ca^2+^ oscillations, which are required for infection thread growth and nodule organogenesis. There are diverse patterns of spatiotemporal Ca^2+^ signals and dynamics, known as Ca^2+^ signatures (Miwa et al. 2006; McAinsh and Pittman 2009).

Following the elucidation of the salinity-induced alterations in *Vicia faba* germination, growth and metabolism, the possible antagonistic or protective effects of calcium against stress was pursued. Calcium has ubiquitous occurrence and interferences in structure and metabolism of plant cells: normal or stressed, in addition to improving soil properties. Accordingly, calcium could, at least partially, restore the physiological activity that has been impaired by the stress imposed (Tester and Davenport 2003). Similarly in this work Na^+^/Ca^2+^ ratio of 1/5, enhanced growth of faba shoots and roots by reversing the NaCl-induced impairments in membranes. Calcium usually interferes by counteracting the stress-induced injuries (e.g., to membranes), and thereby energy consumption for maintenance processes is saved. Membrane stability index and injury was also disordered by salinity, while well ordered by calcium. NaCl toxifies plants by the toxicity effect of Na^+^ and Cl^−^ ions, e.g., via triggering membrane injury and subsequent leakage of metabolites (Jia et al. 2014). In this respect, Cramer et al. (1990) reported that high Ca^2+^ increased the osmolality for two maize cultivars; Na^+^ and Ca^2+^ were the principal solutes involved in osmotic adjustment with minor increases in K^+^ and soluble sugars. The addition of Ca to ameliorate the harmful effects of NaC1 has been highlighted by La Haye and Epstein (1971). Externally supplied Ca^2+^ reduces salt toxicity presumably by facilitating higher K^+^/Na^+^ selectivity (Cramer et al. 1985; Liu and Zhu 1997). Therefore, applying Na/Ca combinations might have improved soil characteristics, of which availability of nutrients to *V. faba* plants in this work. Ca^2+^ is warranted as a second messenger and function in signal transduction of many stimuli. An increase of cytosolic Ca^2+^ in response to salt potentiates stress signal transduction and leads to salt adaptation (Mendoza et al. 1994). Calcium triggered water influx into tobacco cells; the Ca^2+^ depleted cells preserved only about half (54%) relative to the control (Ca^2+^-supplemented) water content (Abdel-Basset and Matsumoto 2008)**.** Changes in intracellular free calcium levels for signal transduction is the onset of response to stresses. The calcium-induced calcium release (CICR) is the calcium-mediated abiotic stress signaling in regulating primary root growth of plants (Wilkins et al. 2016; Zhang et al. 2020).

Respiration of *Rhizobium* was indicative for its survival at salinity. Leaves respiration followed, more or less, the same attitude, i.e., inhibition by salinity and enhancement by calcium. Increasing NaCl concentration clearly depressed respiration of both roots and shoots. Leaf respiration rates decreased under salinity in most species, but the decline was always smaller than that of photosynthesis, therefore resulting in decreased photosynthesis to respiration ratio (indicative of shoot carbon balance). The decline in respiration in response to salinity seems to be part of a systemic metabolic cascade, which occurs at conditions where salinity severely restricts CO_2_ availability inside leaf cells, therefore creating the risk of a secondary oxidative stress (Flexas et al. 2008). A response of respiration to salinity is primarily associated with the direct effects of salinity on enzyme function (Walker and Loneragan 1981; Seemann and Critchly 1985). High concentrations of salinity have often been reported to increase respiration: such increase is greater in salt sensitive than salt tolerant species (Semikhatova 1993). Salt inhibited O_2_ uptake of bacteroids isolated from nodules of faba bean; however, salt treatment of plants decreased respiratory capacity in faba-bean bacteroids. Inhibition of ARA under moderate saline stress may be related to the drop in bacteroid respiration (Delgado et al. 1994). Rates of respiration of *Vicia faba* were higher in mesophyll cell protoplasts than epidermal cell protoplasts (Long et al. 2015).

In this work, calcium induced ameliorative response of *Vicia faba* to salinity stress via improving membrane stability, which preserves cellular metabolites from leakage and, thus, served for enhancing growth (fresh and dry mass). Also, nitrogen fixation by *Rhizobium* was improved and supported growth by nitrogenous intermediates despite the presence of salinity.

## Conclusions

Na^+^ toxicity and other inhibitory effects of NaCl on *Vicia faba* plants like water or osmotic and ion imbalance stresses can be overcome, at least partially, by substitution of Ca^2+^ for Na^+^. Ca^2+^ concentration can be optimized to antagonize Na^+^ toxicity. Particularly, Na^+^/Ca^2+^ ratio of 1/5 ameliorated the inhibitory action of NaCl and induced improvements in germination and growth of* Vicia faba* relative to the control or salinized plants. *Rhizobium* addition was also ameliorative via enhancing nodulation and nitrogen fixation.

## Data Availability

The data will be provided on request.
